# High-Intensity Functional Training Guided by Individualized Heart Rate Variability Results in Similar Health and Fitness Improvements as Predetermined Training with Less Effort

**DOI:** 10.3390/jfmk6040102

**Published:** 2021-12-13

**Authors:** Justin A. DeBlauw, Nicholas B. Drake, Brady K. Kurtz, Derek A. Crawford, Michael J. Carper, Amanda Wakeman, Katie M. Heinrich

**Affiliations:** 1 Department of Kinesiology, Kansas State University, Manhattan, KS 66506, USA; bkkurtz@ksu.edu (B.K.K.); mcarper@pittstate.edu (M.J.C.); kmhphd@ksu.edu (K.M.H.); 2 Department of Health, Human Performance, and Recreation, Pittsburg State University, Manhattan, KS 66506, USA; nichbinderdrake@gmail.com; 3 School of Nutrition, Kinesiology, and Psychological Science, University of Central Missouri, Warrensburg, MO 64093, USA; dcrawford@ucmo.edu (D.A.C.); wakeman@ucmo.edu (A.W.)

**Keywords:** autonomic nervous system, exercise intensity, training prescription

## Abstract

Heart rate variability (HRV) may be useful for prescribing high-intensity functional training (HIFT) exercise programs. This study aimed to compare effects of HRV-guided and predetermined HIFT on cardiovascular function, body composition, and performance. Methods: Recreationally-active adults (*n* = 55) were randomly assigned to predetermined HIFT (*n* = 29, age = 24.1 ± 4.1 years) or HRV-guided HIFT (*n* = 26, age = 23.7 ± 4.5) groups. Both groups completed 11 weeks of daily HRV recordings, 6 weeks of HIFT (5 d·week-1), and pre- and post-test body composition and fitness assessments. Meaningful changes in resting HRV were used to modulate (i.e., reduce) HRV-guided participants’ exercise intensity. Linear mixed models were used with Bonferroni post hoc adjustment for analysis. Results: All participants significantly improved resting heart rate, lean mass, fat mass, strength, and work capacity. However, no significant between-groups differences were observed for cardiovascular function, body composition, or fitness changes. The HRV-guided group spent significantly fewer training days at high intensity (mean difference = −13.56 ± 0.83 days; *p* < 0.001). Conclusion: HRV-guided HIFT produced similar improvements in cardiovascular function, body composition, and fitness as predetermined HIFT, despite fewer days at high intensity. HRV shows promise for prescribing individualized exercise intensity during HIFT.

## 1. Introduction

Exercise training programs relying on predetermined volume and intensity often result in heterogeneous fitness outcomes across individuals [1]. To maximize training potential, employing an individualized training program is the most practical applied strategy [2]. An important factor in individualizing training and reducing the risk of maladaptation, is the ability to effectively monitor responses to training stressors [3]. Training stress is often described as the input variable that is manipulated to elicit a desired physiological response and is categorized as either external (e.g., speed, repetitions) or internal (e.g., heart rate, lactate) load [4,5].

A promising, non-invasive tool to monitor internal load to optimize training outcomes is heart rate variability (HRV) [6,7]. HRV is assessed by measuring the time intervals between successive heartbeats, since an increase or decrease in these intervals reflects changes in cardiac autonomic regulation [8]. HRV is a valid tool to assess individual variation in adaptation, fatigue, and overtraining during training programs [7,9,10]. Daily HRV measurements are often used to adjust training prescriptions for endurance activities such as running [9,11], cross country skiing [12], and cycling [6,10]. Endurance training programs utilizing HRV-guided individualization improve VO2peak, peak power in runners [11], and 40 min time trial performance in cyclists [10]. Additionally, resistance training frequency can be increased when using HRV to determine recovery intervals [13].

While these findings are promising, their focus on single modality endurance training regimens does not reflect the complexity of high-level sport training or current trends in exercise programs. High-intensity functional training (HIFT), a “Top 10 Fitness Trend” in 2018, is comprised of functional, multi-joint aerobic and muscle strengthening exercises performed at relative high effort or intensity [14]. HIFT combines components of aerobic, weightlifting, and body weight exercises into training sessions in constantly variable patterns across multiple time domains, creating a unique stimulus virtually every day [15]. This uniqueness of HIFT creates difficulty when attempting to quantify training loads with external markers [15,16]. However, HIFT is inherently individually modified as the exercises, intensity levels, and/or time domains can be adapted as needed for each individual [17]. Thus, HIFT programs are ideally situated to benefit from implementing HRV-guided training prescriptions.

To the best of our knowledge, no study has investigated the efficacy of HIFT exercise programs when guided by daily HRV. The purpose of the current investigation was to determine the effects of HRV-guided HIFT training compared to predetermined HIFT training on cardiovascular function, body composition, and performance outcomes in recreationally active participants. We hypothesized that HRV-guided HIFT (i.e., prescribing training volume and intensity of HIFT in response to daily HRV status) would result in reduced training volume at high intensity and improved fitness outcomes compared to predetermined HIFT training.

## 2. Materials and Methods

### 2.1. Experimental Design

This study was an 11 week, two-site prospective randomized controlled trial intervention, designed to determine the efficacy of HRV as means to modulate HIFT. Participants were randomly assigned at each site to either an experimental (HRV-guided) or control group (predetermined), with groups balanced for sex. After assignment, both groups completed 14 days of resting HRV measurements, which served as baseline values. Following the baseline period, participants continued taking morning HRV readings and began the exercise intervention which consisted of two three-week training blocks interspersed with pre- and post-intervention testing weeks; a mid-point week was used to recalibrate HRV metrics. Table 1 illustrates the study timeline. During training weeks, participants completed 60-min HIFT sessions on five consecutive days (Monday–Friday) followed by two days of recovery (Saturday and Sunday). Participants were asked to participate in 30 total training sessions with multiple training times available during the training intervention so as to maintain an appropriate participant-to-researcher ratio and accommodate schedules. The HRV-guided group had exercise intensity and volume modulated based on their morning HRV values, while the predetermined group completed training as prescribed. Performance measurements were assessed with participants attending two laboratory sessions during testing weeks, with 48 h of rest in-between.

### 2.2. Participants

Fifty-five recreationally active men and women aged 18–35 years were recruited for this study. Participant baseline characteristics sorted by group and sex are presented in Table 2. All participants had been exercising regularly, while not pursuing any specific health or fitness-related goal (e.g., weight loss or competition preparation) for at least six months at the time of enrollment for this study. All participants reported no physical or health limitations for vigorous exercise, as determined by a medical health history questionnaire and physical activity readiness questionnaire [18]. In addition, no participants indicated a health condition or medication that would alter cardiac rhythms. Written informed consent was obtained from all participants prior to study commencement. The study was performed in accordance with the Declaration of Helsinki, and two university institutional review boards approved all procedures (IRB #9131).

### 2.3. Procedures

#### 2.3.1. Heart Rate Variability

All participants took daily morning HRV readings using a commercially available smartphone application for both iOS and Android (Amsterdam, The Netherlands; see http://www.hrv4training.com/ (1 June 2021)). The HRV4Training software utilizes photoplethysmography to determine the variability in R-R intervals from continuous heart rate data [19,20]. To maintain HRV reliability, participants were instructed to use the application in the morning upon waking, after excretion from the urinary bladder and resting for five minutes. To perform readings, participants placed their index finger over the smartphone camera for one-minute while in the supine position [21]. The HRV4Training application has a built-in methodology for signal filtering, processing, interpolation, artifact correction, and R-R peak detection which can be found in the reference for the application development [19]. For day-to-day monitoring of individual recovery (i.e., sympathovagal balance) HRV was measured as the root mean squared of successive differences (RMSSD). Due to the lack of normality, the RMSSD was transformed using the natural logarithm (LnRMSSD), which was then multiplied by two so that LnRMSSD (HRVdaily) could be viewed on a scale of approximately one to ten for ease of interpretation and to reflect the application display [22].

#### 2.3.2. Resting Heart Rate

Participant resting heart rate (rHR) was collected daily simultaneously with morning HRV readings using photoplethysmography via the HRV4Training smartphone application.

#### 2.3.3. Coefficient of Variance of Heart Rate Variability

Participant coefficient of variation in HRV (CV of HRV), the amount of day-to-day variability in HRV scores, was collected simultaneously with morning HRV readings using photoplethysmography via the HRV4Training smartphone application [23].

#### 2.3.4. Body Composition

Body composition was measured for all participants at pre- and post-testing. Participant height was measured to the nearest 0.1 cm with a Charder stadiometer (Model HM 200P; Taichung City, Taiwan) at both sites. Weight was measured to the nearest 0.1 kg via a Tanita (Tanita TBF-140, Tokyo, Japan) at site 1 and Tanita TBF310 bioelectrical impedance scale (Arlington Heights, IL, USA) at site 2. Body fat percentage (BF%), fat mass (FM), and lean mass (LM) were measured using a dual energy x-ray (DEXA; Discovery A QDR, Hologic, Inc., Marlborough, MA, USA) at site 1 and a Tanita TBF310 bioelectrical impedance scale at site 2.

#### 2.3.5. Aerobic Capacity

Aerobic capacity was determined as maximal oxygen consumption (VO_2_max) via the Bruce treadmill test protocol [24]. Site 1 used a predictive-regression equation based upon time to exhaustion [25] to determine aerobic capacity; the standard error of the estimate for males was ±3.55 mL/kg/min and ±2.70 mL/kg/min for females. Site 2 completed the Bruce treadmill test protocol, followed by a maximal oxygen consumption validation to ensure there was no further increase in oxygen consumption with increasing workload [26]. Expired gas fractions were assessed through breath-by-breath data recording, and measurements were analyzed at 15 s intervals (ParvoMedics TrueOne 2400 Metabolic, Salt Lake City, UT, USA). The gas calibration and metabolic cart flow were calibrated before each testing session using a 3 L syringe and following manufacturer instructions. Heart rate was recorded continuously using a Polar H7 chest strap heart rate monitor (Polar Electro OY, Kempele, Finland).

#### 2.3.6. Physical Work Capacity

Physical work capacity was measured through a 10 min workout in which participants completed as many rounds as possible of the following: 12 goblet squats (20 kg kettlebell for men, 12 kg kettlebell for women), 12 burpees, and 24 calories on a rowing ergometer (Model D, PM5 Monitor, Concept 2 Inc., Morrisville, VM, USA).

#### 2.3.7. Muscular Strength

Maximal strength was determined by the one-repetition maximum (1RM) protocol for the barbell back squat, barbell overhead (OH) press and barbell deadlift in kilograms [27], in accordance with previous research methodology [15]. Individual sum totals for 1RM for back squat, OH press, and deadlift were designated as each participant’s CrossFit Total (CFT) [28]. Each lift was supervised and verified by certified exercise professionals, who were also research assistants, and participant rest times were controlled with a minimum of three minutes and a maximum of five minutes between maximal attempts [29].

#### 2.3.8. High-Intensity Exercise Training Program

The high-intensity exercise program employed in this study was HIFT, utilizing a popular, community-based HIFT template [30]. All training sessions were performed indoors as group exercise and supervised by a research assistant holding a CrossFit^®^ Level 1 certificate. Training sessions for site 1 were held at a community HIFT facility while sessions for site 2 were held within the Functional Intensity Training Lab at Kansas State University. The training protocol for this program has been previously described by Crawford et al. [15], and specific details of the structure and components for each daily training session can be found in Table A1. All training sessions lasted approximately 1 h and consisted of an instructor-led warm-up, movement preparation period, daily workout, and cool-down. A total of 30 training sessions were programmed, and an adherence rate of 80% was required for participant inclusion in data analysis. Participants remained in free-living conditions and were asked to not engage in any additional exercise training outside of the study.

### 2.4. Modulation for High-Intensity Exercise Training Program

A 14 day baseline period was used to establish individual baseline HRV values. Individual seven-day rolling averages (Ln rMMSD7day) were calculated to determine and track shifts in resting HRV in response to the training. The Ln rMMSD7day was used as it has been demonstrated to be superior in predicting training stress rather than single-day HRV values [7]. Smallest worthwhile change (SWC) windows were set to monitor meaningful changes from baseline HRV. Previous investigations have established the SWC in resting HRV as ±0.5 standard deviation from an individual’s mean Ln rMSSD [2,7,11,31]. For this study, two SWC change windows were calculated as ±0.5 standard deviation (SWC1) and ±1 standard deviation (SWC2) from the individual’s mean Ln rMMSD, in order to modulate training stress during the exercise intervention.

Each HRV-guided participant was prescribed reduced training volume and intensity when their rolling seven-day average of HRVdaily (HRV7day as indexed by Ln rMMSD7day) differed meaningfully from baseline values such that it fell within a SWC window [32]. When a participant’s Ln rMMSD7day was within the SWC1 no training modifications were prescribed. If participant’s Ln rMMSD7day fell between SWC1 and SWC2, their scheduled workout was reduced 25% in volume (i.e., repetitions) and external load (i.e., absolute weight). If the participant’s Ln rMMSD7day exceeded the SWC2, they completed a low-intensity (i.e., >50% HRR) active recovery session (e.g., walking and light stretching activities) for a fixed duration of 20 min. A detailed description of the modified and light training session is provided in Table A2. The HRV values obtained during the baseline period were used for the first block of training. After the pre-intervention testing and three weeks of training (15 training sessions) HRV means and both SWC monitoring windows were recalculated for the second training period, as previous findings have demonstrated how changes in fitness may alter resting HRV [33,34,35], and the dose of completing 15 HIFT sessions should be sufficient to elicit fitness improvements [16,36,37]. The Predetermined group completed all training sessions without intensity modulation. Site 1 assessed Edward’s training load to evaluate the efficacy of the modulated training, which is available in previously published research [32].

### 2.5. Statistical Analyses

Data were analyzed using the R statistical computing environment and language [38] via the Jamovi graphical user interface [39]. Data were checked for normality using the Shapiro-Wilk test and visual inspection of the corresponding Q-Q plots of residuals. Relationships between fixed effects (i.e., group and timepoint) and outcome metrics (i.e., cardiovascular, body composition, and performance) data were assessed using linear mixed-effects models via the GAMLj General Analysis for Linear Models module [40]. Individual participants were input as random factors within the models and lean body mass was used as a covariate, due to significant correlations identified with outcomes metrics. An alpha level of 0.05 was used for all statistical inferences. Post hoc assessments were adjusted using the Bonferroni correction. Effect sizes (ES) were calculated for within and between group changes. ES were classified as 0.2 “small”, 0.5 “medium”, and 0.8+ “large” [41].

## 3. Results

**Figure 1:**
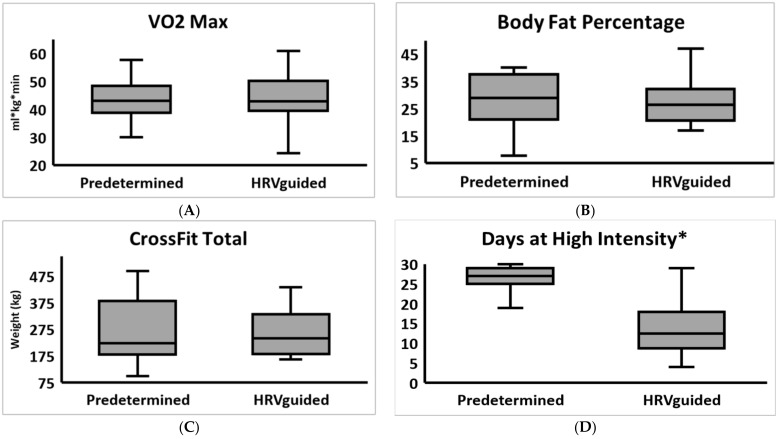
Changes in primary outcome metrics (**A**) VO_2_max, (**B**) body fat percentage, (**C**) CrossFit total, and (**D**) days at High Intensity sorted by group. * Indicates a statistically significant difference.

Baseline and post-test values for each training group are shown in Table 3. The HRV-guided training resulted in similar changes in cardiovascular function, body composition, and performance as the predetermined training (Table 3 & Figure 1). The greatest percentage changes were for predetermined BF% (15.7% decrease) and FM (15.1% decrease), and HRV-guided squat (14.2% increase) and deadlift (12.6% increase). The HRV-guided group completed significantly fewer days at high intensity (DHI) than the predetermined group, as shown in Table 4. Participants displayed a high training and daily HRV monitoring adherence (Table 4).

### 3.1. Effects on Cardiovascular Function

A significant main effect for time was observed for HR (F = 4.89; mean difference = −3.25 ± 1.47 bpm; 95% CI = −6.14, −0.37; *p* = 0.035) with a reduction in resting HR being observed across both conditions from pre- to post-test. No other main effects on cardiovascular function were observed.

### 3.2. Effects on Body Composition

Significant main effects for time were observed for LM (F = 16.43; mean difference = 1.19 ± 0.29 kg; 95% CI = 0.61, 1.77; *p* < 0.001) and FM (F = 4.39; mean difference = −0.62 ± 0.3 kg; 95% CI = −1.12, −0.36; *p* = 0.045), with all individuals improving LM and FM at post-test. No other main effects on body composition were observed.

### 3.3. Effects on Performance Outcomes

Significant main effects for time were observed for work capacity (F = 14.92; mean difference = 16.87 ± 4.37; 95% CI = 8.31, 25.44; *p* < 0.001), squat (F = 29.16; mean difference = 7.98 ± 1.48 kg; 95% CI = −5.08, 10.87; *p* < 0.001), OH Press (F = 10.52; mean difference = 2.62 ± 0.81 kg; 95% CI = 1.04, 4.20, *p* < 0.003), deadlift (F = 22.09; mean difference = 10.37 ± 2.21 kg; 95% CI = 6.05, 14.70; *p* < 0.001), and CFT (F = 20.68; mean difference = 21.79 ± 4.18 kg; 95% CI = 13.61, 29.88; *p* < 0.001) where both groups improved at post-test. No other main effects on performance outcomes were observed.

### 3.4. Effects on Intervention Metrics

A significant main effect for group was observed for DHI (F = 270.46; mean difference = −13.56 ± 0.83 days; 95% CI = −15.20, −11.99; *p* < 0.001) with the HRV-guided group training fewer DHI. Training adherence to the 30 prescribed training sessions for the Predetermined group was 26.3–27.6 sessions and for the HRV-guided group was 25.3–26.7 sessions.

## 4. Discussion

This study tested the effects of HRV-guided and predetermined HIFT on health and fitness outcomes in recreationally active participants. Our results support our first hypothesis, as HRV-guided prescription resulted in fewer DHI compared to a predetermined prescription. This is demonstrated by the HRV-guided group completing 17 of 30 days as modulated, lower intensity training days. Our second hypothesis that HRV-guided prescription would elicit greater improvements in fitness outcomes than the predetermined group was not supported by the data. This is evident through lack of significant differences between groups for changes in all primary outcome fitness measures. Collectively, these findings are of interest as they demonstrate that HRV-guided training results in similar improvements across fitness outcomes while spending fewer training sessions at high intensity compared to a predetermined prescription.

Our finding that HRV-guided training did not result in greater changes in aerobic or work capacity than predetermined training in a 9-week HIFT program was similar to previous aerobic exercise investigations where the HRV-guided group displayed increases in aerobic capacity with no significant difference between groups [6,10,11]. Additionally, neither a small or moderate effect size was observed between groups as previously reported by Vesterninen et al. [42] and Nuuttila et al. [43], respectfully. This finding is not atypical as Hautala et al., [44] has shown that aerobic capacity adaptations are not universal and may be driven by intrinsic factors that predispose individuals to favorable adaptations based on training mode.

We observed no significant differences between groups on improvements in maximal squat, OH press, deadlift, and CrossFit total. The lack of observed group differences is similar to the findings of De Oliveira et al. [13] on maximal strength in young resistance-trained men undergoing HRV-guided training. However, De Oliveria et al. [13] used HRV to augment training frequency, while we used HRV to modulate training intensity. Our findings extend those of De Olivieria et al. [13] and suggest that HRV is a practical tool to individualize the prescription of training frequency and intensity. This enhances the practitioner/coach’s ability to determine when and how much stress to apply in training.

Our participants showed an increase in overall strength following HIFT participation regardless of group. The finding that HIFT is a valid program structure for improving strength is supported by the findings of Heinrich etl al. [45] and Buckley et al. [46] in which HIFT participants displayed increases in bench press, back squat, OH press and deadlift 1RM. It is possible that the observed changes in strength were a result of our participants being classified as “novice”, or as a result of an effective training paradigm. In order to determine the cause, future investigations need to apply this intervention across different experience classifications of HIFT participants. These findings demonstrate that HIFT 5 days/week-1 is an effective methodology for improving muscular strength.

Morning rHR significantly decreased for HRV-guided and Predetermined groups from pre- to post-test, whereas no significant changes were observed in HRV or the CV of HRV. Our findings conflict with those of Kliszczewicz et al. [47] who did not observe improvements in rHR after 15 weeks of HIFT, although they also did not find change in HRV. The lack of observed change in HRV may be a function of the nature of HIFT, as Schneider et al. [48] observed a decrease and no change in HRV following a microcycle of strength training and high-intensity interval training, respectively. Although non-significant, changes were found; we did observe a trend for increases in HRV suggesting an increase in parasympathetic activity. Previously, it has been demonstrated that increases in parasympathetic activity are associated with improved fitness characteristics as well as reduced homeostatic perturbations in response to subsequent stressors [7,49,50].

Of note we observed similar fitness improvements in both groups despite the HRV-guided group spending significantly less time training at high-intensity, namely, 13 less days. This is consistent with the findings of Vesterinen et al. [42] in which HRV-guided recreational endurance runners spent less time training at moderate and high intensity. Since an individual’s HRV response or ability to maintain homeostatic balance can vary due to training history, exercise modality and exercise intensity, a predetermined training prescription may under- or over-estimate the necessary recovery time required [13,51,52,53]. The use of an HRV-guided training prescription may aid practitioners/coaches in optimizing the timing of training stress application.

In addition, participant body composition improved in both of our training groups. This finding contrasts with those of Nuuttila et al. [43] in which no changes in body weight or fat percentage were observed after 11 weeks of HRV-guided running. The changes we observed may be attributed to the high levels of body fat of our participants at >22% versus < 13% for Nuuttila et al.’s [43] participants. Favorable changes in body composition were also found by Feito et al. [54] in both men and women, following 16 weeks of HIFT. As in Feito et al.’s [54] study, our participants were not engaged in physical activity specifically targeting changes in body composition prior to the study, thus allowing for a significant change in body composition from pre- to post-test as a result of the training intervention.

A limitation of this study is that HRV measurements were taken by the individual participants and not within a lab setting, which impairs the standardization process. We are unable to say with certainty that all HRV measurements were protocol adherent throughout the study. A degree of inherent trust must be allotted to participants to strictly adhere to the measurement protocols, and while this increases the external validity of our findings it may have affected our internal validity. We were unable to record and quantify participant internal load (e.g., rHR) or external load (e.g., total training volume) during each training session as was done by De Oliveira et al. [13], to demonstrate the difference in the total work completed by both groups. Quantifying the total work completed by participants within each group would provide additional support to the reduced training load completed by the HRV-guided group. Due to the two-site design, different measures of body composition were used at each site, which may contribute to an increased variability in this outcome metric. Participants were instructed to refrain from engaging in any additional exercise outside of the intervention, yet we were unable to ensure these instructions were adhered to throughout the study period. Finally, we were unable to determine the contribution of muscular hypertrophy to the strength gains observed, as muscle cross-sectional area was not assessed.

A key strength of our study is that we were able to demonstrate how a commercially available smartphone application, with a low individual cost, can be an effective tool for modulating the individual prescription of exercise intensity. Additionally, we demonstrated high adherence to daily HRV recordings and the exercise protocols by participants. This demonstrates that daily HRV recordings are manageable for participants over a period of 11 weeks. Our participants displayed a high level of HRV compliance (>90%) and training adherence (>80%) over the 11 week intervention. Finally, this is the first study to modulate HIFT training prescription based on individual HRV.

## 5. Conclusions

In conclusion, modulating HIFT exercise intensity by individual HRV status, among recreationally active participants, resulted in similar fitness improvements as predetermined HIFT for aerobic capacity, strength, cardiovascular adaptations, and body composition, despite spending fewer days training at high intensity. Practically, our findings suggest that the use of a rolling average of HRV is an effective tool for modulating daily training intensity, with a focus on individual prescription. Coaches and practitioners can use HRV as a tool to effectively individualize exercise prescription for HIFT participation, although additional research is needed to examine the effects for well-trained participants.

## Figures and Tables

**Table 1:** Study timeline from baseline to post-testing.

Study Duration 11 Weeks						
Weeks 1–2	Random Assignment	Week 3	Weeks 4–6	Week 7	Weeks 8–10	Week 11
Baseline HRV & randomization	HRV-guided	Pre-testing VO_2_max, strength & body composition	HRV- modulated training	Mid-point recalibration of HRV SWC windows	HRV- modulated training	Post-testing VO_2_max, strength & body composition
	Predetermined		Predetermined training		Predetermined training	

**Table 2:** Participant descriptives by group * sex.

	Men (HRV-Guided) (*n* = 12)	Men (Predetermined) (*n* = 14)	Female (HRV-Guided) (*n* = 12)	Female (Predetermined) (*n* = 17)
Age (years)	25.0 ± 5.1	23.3 ± 2.8	22.4 ± 3.4	24.6 ± 4.8
Weight (kg)	83.4 ± 10.8	89.8 ± 15.5	72.5 ± 21.9	71.8 ± 9.6
Height (cm)	181 ± 8	182 ± 6	164 ± 5	165 ± 4

**Table 3:** Within and between group comparisons of pre- and post-test changes in key outcomes.

	HRV-Guided				Predetermined				Between Group
	Pre	Post	% Change	ES	Pre	Post	% Change	ES	ES
Cardiovascular function									
Resting heart rate (bpm)	73.6 ± 9.8	69.3 ± 9.0	−5.84	0.46	74.6 ± 14.6	72.7 ± 11.4	−2.55	0.15	0.33
Heart rate variability (ms)	8.4 ± 1.1	8.6 ± 1.1	2.38	0.14	8.7 ± 1.2	8.7 ± 1.2	0	0.01	0.09
CV of HRV (ms)	10.1 ± 3.9	9.0 ± 3.8	−10.89	0.28	8.7 ± 3.3	9.5 ± 3.1	9.20	−0.24	0.14
Body composition									
Body fat %	31.8 ± 11.1	29.2 ± 9.7	−8.18	0.63 *	31.8 ± 8.3	26.8 ± 8.1	−15.73	0.61 *	0.27
Lean mass (kg)	54.5 ± 13.5	54.8 ± 13.3	0.55	0.02	52.6 ± 11.2	54.0 ± 11.5	2.66	−0.12	0.06
Fat mass (kg)	23.9 ± 8.8	23.5 ± 8.7	−1.67	0.05	23.9 ± 8.8	20.3 ± 8.5	−15.06	0.42	0.37
Fitness outcomes									
VO_2_max (mL * kg * min)	42.1 ± 6.8	43.0 ± 7.5	2.14	0.13	44.4 ± 6.4	44.2 ± 8.0	−0.45	0.03	0.15
Work capacity (reps)	131 ± 36	147 ± 35	12.21	0.45	127 ± 24	145 ± 26	14.17	−0.70 *	0.06
Squat (kg)	90.2 ± 44.5	103 ± 45.0	14.19	0.29	87.6 ± 33.2	99.1 ± 31.5	13.13	−0.36	0.10
Press (kg)	41.6 ± 18.9	45.3 ± 21.4	8.89	0.18	41.5 ± 16.2	45.5 ± 16.4	9.64	−0.25	0.01
Deadlift (kg)	103 ± 46	116 ± 47	12.62	0.27	107 ± 34	121 ± 47	13.08	−0.34	0.11
CrossFit total (kg)	232 ± 109	259 ± 108	11.63	0.25	237 ± 82	266 ± 85	12.24	−0.35	0.07

Values are presented as mean ± SD. * moderate effect size. VO_2_max, maximal oxygen consumptions; ES, effect size.

**Table 4:** Study intervention metrics by group.

	HRV-Guided		Predetermined		Between Group
	Mean	95% CI	Mean	95% CI	ES
Days at high intensity (days)	12.9 ± 5.6	11.7; 14.1	26.5 ± 2.6	25.4; 14.1	3.12 **
Training adherence (days completed)	86.8 ± 9.5	84.3; 89.0	89.8 ± 7.6	87.5; 92.0	0.35
HRV compliance (% days recorded)	95.1 ± 4.8	93.8; 96.7	94.6 ± 5.9	92.9; 95.6	0.09

Values are presented as mean ± SD. ** large effect size. CI, confidence interval; ES, effect size.

## Data Availability

The data presented in this study are available on request from the corresponding author.

## Appendix A

**Table A1:** Detailed description of daily high-intensity functional training intervention.

Day	Structure	Structured Daily Workout
1	M	Two-mile Run (no time cap)
2	GW	[8 Push Press (135/95 lbs.) + 8 Pull-Ups] × 5 rounds for time
3	MGW	[12 Goblet Squats (45/25 lbs.) + 12 Burpees + 24 Calorie Row] AMRAP in 10 min
4	MG	[400-m Run + 25 Box Jumps (18/12″)] × 3 rounds for time
5	W	Deadlift 5-5-5-5-5 working up to target 85% of 1RM
6	G	Kipping Pull-Up practice for 20 min
7	WM	[10 Thrusters (135/95 lbs.) + 100 Double Unders] × 4 rounds for time
8	GWM	[6 Handstand Push-Ups + 12 Deadlifts (185/135 lbs.) + 500 m Row] AMRAP in 12 min
9	GW	[15 Ring Rows + 20 Wall Balls (20/14 lbs.)] × 4 rounds for time
10	M	8 km Partner Row (no time cap)
11	W	Front Squat 1-1-1-1-1-1-1-1-1-1 working up to target a 1RM
12	MG	[400-m Run + 20 Push-Ups] × 5 rounds for time
13	WMG	[5 Cleans (135/95 lbs.) + 10 Pull-Ups + 15 Double Unders] AMRAP in 15 min
14	WM	[10/20–8/16–6/12–4/8–2/4 repetitions of Power Clean/Calorie Row] for time
15	G	Handstand Push-Up Practive for 20 min
16	W	Squat 3-3-3-3-3-3-3 working up to target 90% 1RM
17	MG	[800-m Run + 25 Sit-Ups] × 3 rounds for time
18	MGW	[50 Double Unders + 5 Box Jumps (18/12″) + 15 Ball Slams (20/14 lbs.)] AMRAP in 15 min
19	GW	[6 Strict Pull-Ups + 6 Front Squats (50% Squat 1RM)] × 4 rounds for time
20	M	Two-mile Run (no time cap)
21	M	Tabata Double Unders × 2
22	GW	[Maximum repetitions Handstand Push-Ups + 6 Deadlifts (75% 1RM)] × 5 rounds for time
23	GWM	[20 Sit-Ups + 16 Dumbbell Clean and Jerk (45/20 lbs.)]
24	WM	[30 Kettlebell Swings (45/20 lbs.) + 400 m Run] × 5 rounds for time
25	G	Strict Pull-Up Practice (Loaded) for 25 min
26	G	Muscle Up Practice for 25 min
27	WM	[6 Squats (50% 1RM) + 50 Double Unders] × 4 rounds for time
28	WMG	[12 Goblet Squats (45/25 lbs.) + 12 Burpees + 24 Calorie Row] AMRAP in 10 min
29	MG	[400-m Run + 10 Handstand Push-Ups] × 5 rounds for time
30	W	Clean 1-1-1-1-1-1-1-1-1-1 working up to target 1RM

M = monostructural (i.e., a single cardiovascular exercise modality) exercise, G = gymnastics exercise, W = weightlifting exercise, and AMRAP = “as many rounds as possible”. Daily workouts were scaled to match individual capabilities on an as-needed basis. All scaling options were in accordance with outlined CrossFit scaling practices as per Glassman (2016) (p. 75). Table is adapted from Crawford et al. (2019).

**Table A2:** Detailed description the modified and light training.

Day	Modified Training	Light Training
1	1.5 m	WALK 20 min
2	25% Volume Reduction. 115/65#	Barbell Press/DB/PVC Pending Strength Levels, DEAD hang stretch or lat banded distraction
3	NO MODULATION	NO MODULATION
4	300 m/Walk 100 m & 18 Jumps 16/12″ Step up	Walk 400 m, 2/Leg/Rd Sampson Stretch
5	5RM Load with 2 RIR “5 × 3” at 85%	5 × 3 at 40% 1RM
6	3 Strict Pull ups, Kipping practice (no kipping pull-ups)	Shoulder-strengthing exercise, light lat pull down machine (RPE LIGHT)
7	8 Thrusters, 150 Single Unders. 115/65	Barbell, DB, PVC thrusters and hopping in place, no rope, 20 min
8	8 min: 4 pushups, 8 deadlifts 135/95, 375 row, RPE 13–17	20 min: Pushups, BB/DB/PVC deadlifts, 400 m walking
9	3 RFT, 16/10	Ring Rows, childs pose, walking, 20 min
10	6 K partner row, not for time or 3 k row if solo	Walking 20 min
11	85% of Back Squat, 10 × 1, 2 RIR	KB/Goblet Squat/Barbell Squat, Walking
12	200 m run, walk back, 15 pushups	20 min: 400 m walk, knee pushups
13	5 Deadlifts, ring rows, 15 single unders	Med Ball Cleans, childs pose, shoulder exercise, walking, 20 min
14	4 rounds, 2 min break b/t: 115/65 6 power cleans, 12 cal row	Med ball cleans, walking 20 min RPE 6–13
15	HS holds	push-ups, walking
16	7 × 3 2 RIR, 75% target	40% 7 × 3
17	600 m Run, 200 m walk. 20 Situps	20 min Walk
18	12 min	20 min Walk
19	3 RFT	20 min: Goblet Squats, Lat Pulldowns
20	1.5 m Run	20 min Walk
21	1.5 Tabata Rounds	20 min Walk
22	4 RFT Max Push ups 2 RIR, 6 Deadlift 50%	20 min: Push ups, Handstand holds, deadlifts, hamstring curls
23	15 min	20 min Stretching
24	4 RFT 28 KB Swings, 200 M run, 200 walk	20 min Walk
25	30 Pull-Ups	Lat Pull-Downs, Ring Row
26	30 Pull-Ups, 20 dips	Lat Pull-Downs, Ring Row, Press
27	3 RFT	20 min: Air Squats, Box Jumps, walking
28	Testing Day, no modifications	Testing Day, no modifications
29	4 RFT, 200 m run, 200 m walk. 9 push ups	20 min Walk
30	7 × 1 front squat, 2 RIR	20 min: Goblet squat, front rack mobility, barbell front squat
